# Simple and Objective Prediction of Survival in Patients with Lung Cancer: Staging the Host Systemic Inflammatory Response

**DOI:** 10.1155/2014/731925

**Published:** 2014-03-05

**Authors:** Derek Grose, Graham Devereux, Louise Brown, Richard Jones, Dave Sharma, Colin Selby, David S. Morrison, Kirsty Docherty, David McIntosh, Penny McElhinney, Marianne Nicolson, Donald C. McMillan, Robert Milroy

**Affiliations:** ^1^Beatson Oncology Centre, 1053 Great Western Road, Glasgow G12 0YN, UK; ^2^University of Aberdeen, Aberdeen, UK; ^3^Inverclyde Royal Hospital, Inverclyde, UK; ^4^Queen Margaret Hospital, Dunfermline, UK; ^5^University of Glasgow, Glasgow, UK; ^6^Glasgow Royal Infirmary, Glasgow, UK; ^7^Aberdeen Royal Infirmary, Aberdeen, UK

## Abstract

*Background*. Prediction of survival in patients diagnosed with lung cancer remains problematical. The aim of the present study was to examine the clinical utility of an established objective marker of the systemic inflammatory response, the Glasgow Prognostic Score, as the basis of risk stratification in patients with lung cancer. *Methods*. Between 2005 and 2008 all newly diagnosed lung cancer patients coming through the multidisciplinary meetings (MDTs) of four Scottish centres were included in the study. The details of 882 patients with a confirmed new diagnosis of any subtype or stage of lung cancer were collected prospectively. *Results*. The median survival was 5.6 months (IQR 4.8–6.5). Survival analysis was undertaken in three separate groups based on mGPS score. In the mGPS 0 group the most highly predictive factors were performance status, weight loss, stage of NSCLC, and palliative treatment offered. In the mGPS 1 group performance status, stage of NSCLC, and radical treatment offered were significant. In the mGPS 2 group only performance status and weight loss were statistically significant. *Discussion*. This present study confirms previous work supporting the use of mGPS in predicting cancer survival; however, it goes further by showing how it might be used to provide more objective risk stratification in patients diagnosed with lung cancer.

## 1. Introduction

Within Scotland lung cancer remains the commonest cause of cancer related death [1]. The prognosis is bleak with the median survival in advanced disease around four months from diagnosis [1]. Survival compares unfavourably with other European countries and the USA [1, 2]. It has often been felt that the Scottish lung cancer population has more comorbidity with poorer performance status thus presenting fewer tolerable therapeutic options.

It is difficult to quantify the complex nature of patient frailty to provide a degree of objective assessment of fitness [3] and as a result prediction of survival in patients diagnosed with lung cancer remains problematical. Currently, prognosis is based upon a combination of stage of disease and performance status although other factors such as weight loss have been identified in the advanced cancer setting [4–6]. However, these host factors (weight loss and performance status) included in clinical decisions are recognised to be subjective in nature.

Recent work shows that the effect of systemic inflammation is detrimental in terms of outcome in cancer in general [7, 8] and in lung cancer specifically [9–16]. The combination of C-reactive protein and albumin when combined to calculate the modified Glasgow Prognostic Score (Table 1) has been previously validated as an independent predictor of survival [17].

The aim of the present study was to examine the clinical utility of this established objective marker of the systemic inflammatory response, the modified Glasgow Prognostic Score (mGPS), as the basis of risk stratification in patients with lung cancer.

## 2. Patients and Methods

Four Scottish centres were included in the study: Aberdeen, Glasgow (Stobhill), Inverclyde, and West Fife. Over a defined period from 2005 to 2008 all newly diagnosed lung cancer patients coming through the regional multidisciplinary meetings (MDTs) were included in the study. In total the details of 882 patients were collected prospectively.

At the time of the patient discussion at the MDT annoymised details were entered into a specifically designed Microsoft Access database. Patient demographics and baseline characteristics (age, sex, postcode, and smoking history), PS (at time of presentation in addition to that six months prior to attendance as estimated by clinicians on questioning patients about fitness), weight loss, laboratory parameters (C-reactive protein, albumin, and ventilatory function), tumour stage and histology, and primary treatment proposed by the MDT were all recorded. If the treatment proposed varied from the 2005 Scottish Intercollegiate Guidelines Network (SIGN) guidelines [18] on lung cancer management the reasons why were also recorded (i.e., age, poor PS, comorbidity, size of tumour, etc.). All four MDTs had input from an oncologist and thoracic surgeon and thus we could be confident of accuracy of the clinical management decision. Details of the study design have previously been published [19].

Information on date of death was obtained via survival analysis undertaken by the Information Service Division (ISD) of NHS Scotland. Death records were complete until 1 June 2011, which served as the censor date for those alive.

### 2.1. Socioeconomic Status

Information on patients' individual educational or occupational social class was not available, and postcode of residence was used to identify the 2006 Scottish Index of Multiple Deprivation (SIMD) ranking as a proxy indicator of their socioeconomic circumstances [20]. The 2006 SIMD is a validated area-based index that uses 37 indicators in seven domains to rank 6505 small geographic areas in Scotland (data zones) from 1 (most deprived) to 6505 (least deprived). These can be subsequently grouped into quintiles, and we used Scottish national quintiles.

### 2.2. GPS/mGPS

A venous blood sample was obtained at diagnosis for measurement of CRP concentration and albumin. The coefficient of variation for these methods over the range of measurement was less than 5%, as established by routine quality control procedures. The GPS was constructed as previously described (Table 1) [21, 22]. In brief, CRP more than 10 mg/L and albumin less than 35 g/dL were each given a score of 1. The GPS was calculated as 0, 1, or 2. Since hypoalbuminaemia alone in the absence of an increased CRP level did not confer a poorer cancer-specific survival in all patients with cancer [8, 22], the GPS was modified to assign a score of 0 in patients with hypoalbuminaemia alone (Table 1) [23].

A number of recent studies have supported the use of mGPS in predicting outcome both in lung cancer and other tumour types [9–17]. As such it was our intention to stratify the group by mGPS and then analyse the impact of more conventional staging methods such as TNM stage and performance status.

### 2.3. Ethics

The audit was discussed with the local ethics committee, and since it was classed as a health service clinical practice audit, formal ethical approval was deemed not to be necessary.

### 2.4. Statistics

All statistical testing was conducted at the 5% level so 95% confidence intervals (CI) are reported throughout. Unless otherwise stated, medians and interquartile range (IQR) are used. The survival time defined as the number of months from study entry until death or if alive at follow-up date was calculated. Univariate survival analysis was carried out using the Kaplan-Meier method and the log rank test. Survival analysis was carried out using Cox's proportional-hazards model and hazard ratios (HR) were calculated. Multivariate survival analysis was performed using a stepwise backward procedure to derive a final model of the variables that had a significant independent relationship with survival. To remove a variable from the model, the corresponding *P* value had to be >0.10.

Statistical analyses were performed using SPSS v19.0 (SPSS Inc., Chicago, IL).

## 3. Results

**Figure 1:**
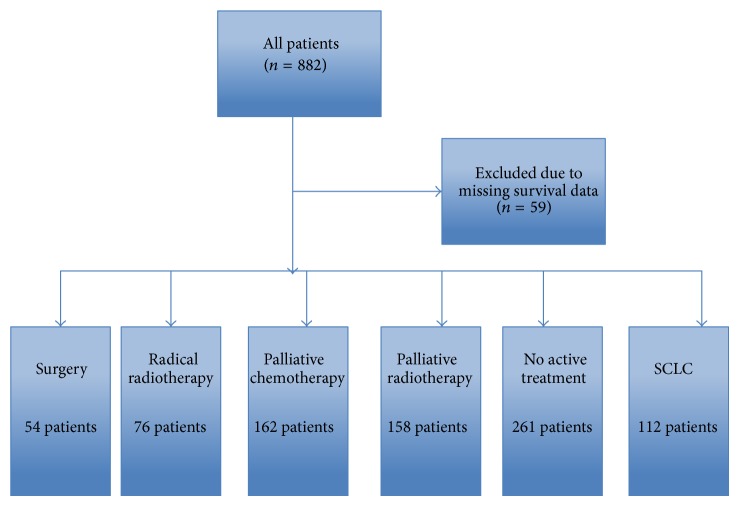
Flow chart of patient selection process.

In total, 882 patients from a number of different treatment groups were included in the study, comprising 297 from Aberdeen, 136 from West Fife, 285 from Glasgow, and 164 from Inverclyde composing; 59 patients were excluded from the study due to missing survival data (Figure 1). Baseline characteristics are shown in Table 2. The median age of participants was 72 years old. The majority were male, current or ex-smokers, and of good performance status with advanced disease and had treatment with palliative intent.

Of the patients 24% were diagnosed on the basis of clinical examination and radiological investigations alone and without histological evidence. This compares favourably with the National Lung Cancer Audit data [24], which had a median rate of 37% of patients who did not have histological confirmation of lung cancer.

Most had an elevated mGPS. The median followup for survivors was 24.5 months (4.6–40.8). The median overall survival was 5.6 months (4.8–6.5). The 12-month survival rate was 30% (SE 2%).

**Figure 2:**
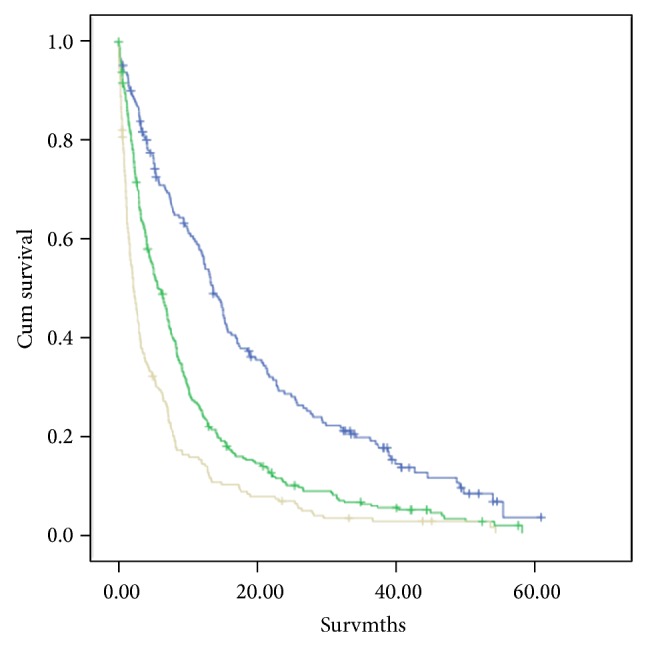
The relationship between GPS (0–2, from top to bottom) and survival. GPS 0 versus 1 (log rank *P* < 0.001), GPS 1 versus 2 (log rank *P* < 0.001).

**Figure 3:**
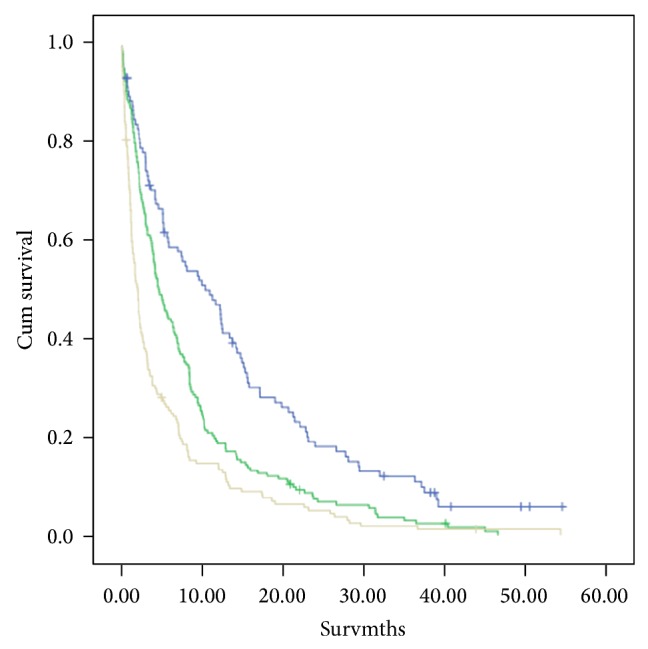
The relationship between mGPS (0–2, from top to bottom) and survival. mGPS 0 versus 1 (log rank *P* < 0.001), mGPS 1 versus 2 (log rank *P* < 0.001).

Survival analysis using both GPS and mGPS was undertaken (Figures 2 and 3). Both were highly significantly associated with survival. Since the mGPS has been most extensively validated and readily extrapolated from previous work using C-reactive protein alone [7, 17], it was used in the remainder of the analysis and to stratify the three groups.

The relationship between the mGPS and clinicopathological characteristics is shown in Table 3. There were 213 patients in the mGPS score of 0 group, 290 patients in the mGPS score of 1 group, and 218 patients in the mGPS score of 2 group. The mGPS was associated with increasing deprivation (*P* < 0.001), pack years smoking (*P* < 0.001), poorer performance status (*P* < 0.001), more weight loss (*P* < 0.001), more advanced disease (*P* < 0.001), more radical treatment (*P* < 0.001), and poorer survival (*P* < 0.001).

The relationship between the clinicopathological characteristics and survival in patients with an mGPS of 0 is shown in Table 4. The median survival was 13.2 (11.2–18.9) months. On univariate survival analysis, performance status (*P* < 0.001), weight loss (*P* < 0.01), stage of NSCLC (*P* < 0.001), radical treatment offered (*P* < 0.01), and palliative treatment offered (*P* < 0.05) were significantly associated with survival. On multivariate analysis, performance status (HR 1.69, 95% CI 1.39–2.06, and *P* < 0.001), weight loss (HR 1.18, 95% CI 1.04–1.33, and *P* = 0.009), stage of NSCLC (HR 1.06, 95% CI 1.01–1.23, and *P* = 0.017) and palliative treatment offered (HR 1.30, 95% CI 1.08–1.55, and *P* = 0.004) were independently associated with survival.

The relationship between the clinico-pathological characteristics and survival in patients with an mGPS of 1 is shown in Table 5. The median survival was 6.1 (4.9–7.3) months. On univariate survival analysis, decreased age (*P* < 0.01), performance status (*P* < 0.001), weight loss (*P* < 0.01), stage of NSCLC (*P* < 0.001), and radical treatment offered (*P* < 0.001) were significantly associated with survival. On multivariate analysis, performance status (HR 1.81, 95% CI 1.55–2.13, and *P* < 0.001), stage of NSCLC (HR 1.08, 95% CI 1.03–1.13, and *P* < 0.01) and radical treatment offered (HR 0.70, 95% CI 0.52–0.94, and *P* < 0.05) were independently associated with survival.

The relationship between the clinico-pathological characteristics and survival in patients with an mGPS of 2 is shown in Table 6. The median survival was 2.1 (1.5–2.7) months. On univariate survival analysis, centre (*P* < 0.01), performance status (*P* < 0.001), weight loss (*P* < 0.001), stage of NSCLC (*P* < 0.001), and radical treatment offered (*P* < 0.01) were significantly associated with survival. On multivariate analysis, only performance status (HR 1.44, 95% CI 1.21–1.71, and *P* < 0.001) and weight loss (HR 1.13, 95% CI 1.00–1.28, and  *P* < 0.05) were independently associated with survival.

The relationship between mGPS, performance status, and survival at 1 year is shown in Table 7. When used in combination survival at 1 year varied from 72% (mGPS 0, PS 0) to 6% (mGPS 2, PS 3). The numbers in the PS 4 subgroup were too small to calculate accurately a survival rate.

The relationship between mGPS, TNM stage (NSCLC patients only), and survival at 1 year is shown in Table 8. Survival varied from 69% (mGPS 0, Stage I NSCLC) to 2% (mGPS 2, Stage IV NSCLC).

To stratify for stage, the relationship between mGPS and PS and survival at 3 months for those patients with advanced NSCLC (St IIIb/IV) is shown in Table 9.

Survival varied from 100% (mGPS 0, PS 0) to 23% (mGPS 2, PS 3). The number of patients in the PS 4 group was too small to accurately calculate survival.

This group was then further stratified to take into account the treatment offered. The relationship between mGPS and PS at 3 months for those patients with advanced NSCLC (St IIIb/IV) undergoing palliative chemotherapy is shown in Table 10. Survival varied from 92% (mGPS 0, PS 1) to 50% (mGPS 2, PS 2). The numbers of patients in the PS 0 and 4 groups were too small to accurately calculate survival.

## 4. Discussion

The results of the present study show for the first time that, in a large cohort of patients with lung cancer and using the mGPS as an objective basis for the prediction of survival, significant factors associated with survival varied significantly. Only performance status and to a lesser extent tumour stage were consistently shown to have independent prognostic value. Furthermore, the combination of the mGPS with either performance status or tumour stage effectively stratified the likely outcome in these patients. Therefore, this simple scheme based on objective criteria provides a new readily applicable approach to the routine clinical evaluation of patients with lung cancer.

In the present study it was of interest that weight loss, a well recognised poor prognostic factor, was inconsistently prognostic when included in the analysis with mGPS and performance status. This may suggest that much of the prognostic value of weight loss is attributable to the activation of the systemic inflammatory response and to the progressive loss of lean tissue leading to nutritional and functional decline [7]. Indeed, activation of the systemic inflammatory response resulted in a marked reduction in median survival of 13 months (mGPS 0) to 2 months (mGPS 2) independent of treatment received. This would suggest that the allocation of treatment was suboptimal and it may be that treatment allocated on a more objective scheme as proposed above will result in improved outcomes in all patients. For example, in those patients with mGPS of 2, neither stage nor treatment had independent prognostic value and therefore it would appear that such poor prognosis patients derive little benefit from standard anticancer treatment. In particular a very honest appraisal of both benefits and toxicities of any treatment should be made with the patient irrespective of their tumour stage [25]. However it must be noted that the very small numbers of patients in these groups (e.g., only 2 patients underwent surgery and 4 underwent radical radiotherapy) make it very difficult to interpret and further studies looking only at radically treatable patients are advised.

The relationship between poor survival and systemic inflammation (the mGPS) remains poorly understood, but it is likely to represent an objective marker of the chronic activation of the innate immune response with the consequent up-regulation of proinflammatory cytokines and growth factors and the resultant cancer cachexia [26–31].

It is clear that, in Scotland, lung cancer continues to confer a very poor outcome with a median life expectancy of approximately 5 months. Even in early disease (TNM I/II NSCLC), with patients undergoing radical treatment with an expectation of cure (10–15% of total number of patients within Scotland [32]) the 5-year survival is only around 30–60% [32]. The advent of more advanced imaging modalities such as PET-CT [18] has improved detection of occult metastasis leading to stage migration and less patients undergoing futile radical local treatment. Nevertheless, the present results highlight the importance of also staging the host systemic inflammatory response. The mGPS is a simple, cheap, and reproducible prognostic tool that has been shown to be a rational starting point for such work.

Since the initial work, a decade ago the combination of C-reactive protein and albumin, the Glasgow Prognostic Score (GPS/mGPS), has been shown to have independent prognostic value in more than 60 studies (>30,000 patients with cancer). This prognostic value has been demonstrated in a variety of clinical scenarios, in particular primary operable cancer [17].

A more recent study of apporximately 2,500 patients [33] and this present study have demonstrated that the mGPS has also clinical utility, together with performance status, in patients with advanced cancer.

In conclusion, the results of the present study confirm the independent prognostic value of the mGPS. In addition, it demonstrates the clinical utility of the mGPS combined with performance status to provide more objective risk stratification in patients diagnosed with lung cancer.

## Figures and Tables

**Table 1:** The Glasgow Prognostic Scores (online only).

	Score
Glasgow Prognostic Score (GPS)	
CRP ≤10 mg/L and albumin ≥35 g/L	0
CRP >10 mg/L or albumin <35 g/L	1
CRP >10 mg/L and albumin <35 g/L	2
Modified Glasgow Prognostic Score (mGPS)	
CRP ≤10 mg/L and albumin ≥35 g/L	0
CRP ≤10 mg/L and albumin <35 g/L	0
CRP >10 mg/L	1
CRP >10 mg/L and albumin <35 g/L	2

**Table 2:** Baseline characteristics of patients with lung cancer (*n* = 882).

Parameter	
Age (years)	72 (31–94) years
Sex	
Male	487 (55%)
Female	395 (45%)
Smoking	
Ever smoked	94%
Mean pack (years)	44 years
Centre	
Aberdeen	294 (34%)
Fife	132 (15%)
Stobhill	245 (32%)
Inverclyde	161 (19%)
Deprivation (quintile)	
Most deprived	267 (30%)
2.00	127 (14%)
3.00	251 (29%)
4.00	175 (20%)
Most affluent	62 (7%)
Performance status	
0	101 (11%)
1	312 (35%)
2	285 (32%)
3	154 (17%)
4	30 (3%)
Stage	
NSCLC I	66 (7%)
NSCLC II	43 (5%)
NSCLC St IIIa	48 (5%)
NSCLC St IIIb	140 (16%)
NSCLC St IV	234 (26%)
SCLC limited	33 (4%)
SCLC extensive	82 (9%)
No histology	210 (24%)
Treatment	
Radical	193 (27%)
Palliative	533 (73%)
mGPS	
0	213 (24%)
1	290 (32%)
2	210 (24%)
Missing data	169 (19%)
12-month survival rate % (SE)	30% (2)

**Table 3:** The relationship between the mGPS and clinicopathological characteristics in patients with lung cancer.

Demographic	mGPS			*P* value (chi-square test)
	0 (*n* = 213)	1 (*n* = 290)	2 (*n* = 218)	
Age				
<60/60–69/70–79/≥80 years	33/71/89/28	44/87/119/54	34/57/91/49	0.064
Sex				
Male/female	115/108	174/130	133/98	0.201
Centre				
Aber/Fife/Stob/Inver	98/10/77/38	146/25/66/67	43/16/126/46	<0.001
Deprivation				
Most-least (quintile)	68/24/71/39/21	75/47/75/77/30	111/21/70/20/9	<0.001
Smoke (pack years)				
NS/<20/20–60/>60	21/24/126/37	11/33/177/69	13/21/128/57	0.014
Performance status				
0/1/2/3/4	42/96/60/22/3	26/130/105/40/3	5/51/88/66/21	<0.001
Weight loss (%)				
0/<5/5–10/>10	147/24/12/38	146/36/13/109	79/45/33/73	<0.001
FEV1 (%)				
<80/61–80/40–60/<40	138/31/45/7	181/48/53/22	130/33/47/17	0.126
Local symptoms				
No/yes	36/127	18/222	29/137	0.246
Tumour stage of NSCLC				
I/II/IIIa/IIIb/IV	32/11/7/38/38	15/19/20/56/84	7/5/12/36/79	<0.001
Tumour stage of SCLC				
Limited/extensive	13/21	10/32	8/18	0.471
Treatment				
Radical treatment				
Surgery/RT/no active	26/9/38	17/15/43	4/2/18	<0.001
Palliative treatment				
Chemo/RT/No active	63/27/49	107/58/55	50/49/75	<0.001
Survival				
Alive/dead	34/181	16/274	8/211	0.003
12-month survival % (SE)	46 (4)	16 (2)	14 (3)	<0.001

**Table 4:** The relationship between parameters and survival in patients with mGPS = 0 (*n* = 213).

Parameter	Patients	Univariate		Multivariate	
	*N*	HR (95% CI)	P value	HR (95% CI)	P value
Age					
<60/60–69/70–79/≥80 years	33/71/89/28	1.16 (0.98–1.37)	0.077	—	
Sex					
Male	115	1	—	—	—
Female	108	0.81 (0.61–1.09)	0.169	—	—
Centre					
Aberdeen	98	1	0.068	—	—
Fife	10	2.72 (1.40–5.29)	—	—	—
Stobhill	77	1.00 (0.72–1.41)	—	—	—
Inverclyde	38	1.13 (0.75–1.70)	—	—	—
Deprivation (most deprived = 1, least deprived = 5)					
1/2/3/4/5	68/24/71/39/21	0.94 (0.85–1.05)	0.299	—	—
Smoking history (NS = never smoker, otherwise pack years)					
NS/<20/20–60/>60	21/24/126/37	0.93 (0.77–1.12)	0.467	—	—
Performance status					
0/1/2/3/4	42/96/60/22/3	1.76 (1.47–2.11)	<0.001	1.69 (1.39–2.06)	<0.001
Weight loss (% body weight)					
0/<5/5–10/>10	147/24/12/38	1.21 (1.08–1.37)	0.002	1.18 (1.04–1.33)	0.009
FEV1 (%)					
>80/80–60/59–40/<40	138/31/45/7	0.94 (0.80–1.12)	0.515	—	—
Local symptoms					
No/yes	36/127	1.38 (0.91–2.09)	0.124	—	—
Stage					
NSCLC					
I/II/IIIa/IIIb/IV	32/11/7/38/38	1.38 (1.25–1.53)	<0.001	1.06 (1.01–1.23)	0.017
SCLC					
Limited/extensive	13/21	1.05 (0.97–1.14)	0.228	—	—
Treatment					
Radical					
BSC/surgery/RT	37/26/8	0.61 (0.48–0.78)	0.001	—	—
Palliative					
Chemo/RT/BSC	59/27/47	1.22 (1.01–1.46)	0.039	1.30 (1.08–1.55)	0.004

**Table 5:** The relationship between parameters and survival in patients with mGPS = 1 (*n* = 290).

Parameter	Patients	Univariate		Multivariate	
	*N*	HR (95% CI)	P value	HR (95% CI)	P value
Age					
<60/60–69/70–79/≥80	33/71/89/28	1.24 (1.09–1.40)	0.001	—	—
Sex					
Male	174	1	0.171	—	—
Female	130	0.84 (0.66–1.08)	—	—	—
Centre					
Aberdeen	145	1	0.425	—	—
Fife	23	0.89 (0.55–1.43)	—	—	—
Stobhill	55	0.99 (0.72–1.36)	—	—	—
Inverclyde	67	1.23 (0.92–1.66)	—	—	—
Deprivation (quintile) (most deprived = 1, least deprived = 5)					
1/2/3/4/5	75/47/75/77/30	0.99 (0.90–1.08)	0.777	—	—
Smoking history (NS = never smoker, otherwise pack years)					
NS/<20/20–60/>60	11/33/177/69	1.02 (0.86–1.22)	0.792	—	—
Performance status					
0/1/2/3/4	26/130/105/40/3	1.83 (1.57–2.14)	<0.001	1.81 (1.55–2.13)	<0.001
Weight loss (% body weight)					
0/<5/5–10/>10	181/48/53/22	1.15 (1.06–1.25)	0.001	—	—
FEV1 (%)					
>80/80–60/59–40/<40	138/31/45/7	0.94 (0.83–1.06)	0.313	—	—
Local symptoms					
No/yes	18/222	1.60 (0.91–2.80)	0.105	—	—
Stage					
NSCLC					
I/II/IIIa/IIIb/IV	15/19/20/56/84	1.31 (1.19–1.45)	<0.001	1.08 (1.03–1.13)	0.002
SCLC					
Limited/extensive	10/32	0.51 (0.23–1.11)	0.091	—	—
Treatment					
Radical					
BSC/surgery/RT	43/17/15	0.55 (0.41–0.72)	<0.001	0.70 (0.52–0.94)	0.017
Palliative					
Chemo/RT/BSC	107/58/55	1.07 (0.90–1.26)	0.436	—	—

**Table 6:** The relationship between parameters and survival in patients with mGPS = 2 (*n* = 218).

Parameter	Patients	Univariate		Multivariate	
	*N*	HR (95% CI)	P value	HR (95% CI)	P value
Age							
<60/60–69/70–79/≥80	34/57/91/49	1.13 (0.98–1.30)	0.084	—	—
Sex							
Male	133	1	0.136	—	—
Female	98	0.81 (0.61–1.07)	—	—	—
Centre							
Aberdeen	43	1	0.002	—	—
Fife	16	1.71 (0.95–3.06)	—	—	—
Stobhill	114	0.64 (0.45–0.92)	—	—	—
Inverclyde	45	0.88 (0.57–1.35)	—	—	—
Deprivation (quintile) (most deprived = 1, least deprived = 5)					
1/2/3/4/5	111/21/70/20/9	1.05 (0.94–1.17)	0.408	—	—
Smoking history (NS = never smoker, otherwise pack years)					
NS/<20/20–60/>60	13/21/128/57	0.83 (0.69–1.00)	0.062	—	—
Performance status					
0/1/2/3/4	5/51/88/66/21	1.51 (1.30–1.76)	<0.001	1.44 (1.21–1.71)	<0.001
Weight loss (% body weight)					
0/<5/5–10/>10	79/45/33/73	1.25 (1.11–1.40)	<0.001	1.13 (1.00–1.28)	0.047
FEV1 (%)					
>80/80–60/59–40/<40	130/33/47/17	0.88 (0.77–1.01)	0.073	—	—
Local symptoms					
No/Yes	29/137	1.51 (0.98–2.33)	0.063	—	—
Stage					
NSCLC					
I/II/IIIa/IIIb/IV	7/5/12/36/79	1.32 (1.15–1.50)	<0.001	—	—
SCLC					
Limited/extensive	8/18	1.01 (0.91–1.12)	0.826	—	—
Treatment						
Radical					
BSC/surgery/RT	18/2/4	0.52 (0.33–0.83)	0.006	—	—
Palliative					
Chemo/RT/BSC	50/49/75	0.92 (0.78–1.10)	0.364	—	—

**Table 7:** The relationship between mGPS and PS and 12-month survival rate (%, SE).

PS	mGPS			Total number of patients
	0	1	2	
0	72% (7)	50% (10)	20% (18)	71
1	65% (5)	31% (4)	30% (7)	259
2	49% (7)	19% (4)	16% (4)	245
3	9% (6)	5% (4)	6% (3)	122
4	NC	NC	NC	20
Total number of patients	212	290	218	—

NC: not calculated where *N* < 10.

**Table 8:** The relationship between mGPS and TNM stage (NSCLC only) and 12-month survival rate (%, SE).

Stage	mGPS			Total number of patients
	0	1	2	
I	69% (19)	28% (12)	NC	62
II	37% (14)	17% (9)	NC	47
IIIa	20% (13)	42% (10)	0%	53
IIIb	20% (6)	6% (3)	14% (5)	154
IV	5% (3)	4% (2)	2% (2)	261
Total number of patients	153	225	173	—

NC: not calculated where *N* < 10.

**Table 9:** The relationship between mGPS and PS and 3-month survival rate (%, SE) in patients with TNM stage IIIb/IV NSCLC (*n* = 374).

PS	mGPS			Total number of patients
	0	1	2	
0	100% (0)	92% (8)	100% (0)	28
1	94% (4)	73% (6)	55% (9)	119
2	65% (11)	62% (7)	43% (8)	105
3	NC	29% (11)	23% (8)	55
4	NC	NC	NC	7
Total number of patients	61	134	111	—

NC: not calculated where *N* < 10.

**Table 10:** The relationship between mGPS and PS and 3-month survival rate (%, SE) in patients with TNM stage IIIb/IV NSCLC and receiving palliative chemotherapy (*n* = 138).

PS	mGPS			Total number of patients
	0	1	2	
0	NC	NC	NC	13
1	92% (7)	74% (8)	54% (14)	60
2	NC	69% (12)	50% (16)	33
3	NC	NC	NC	12
4	NC	NC	NC	0
Total number of patients	27	61	30	—

NC: not calculated where *N* < 10.
